# Hologram imaging quality improvement by ionization controlling based on the self-trapped excitons with double-pulse femtosecond laser

**DOI:** 10.1515/nanoph-2022-0379

**Published:** 2022-10-24

**Authors:** Feifei Wang, Lan Jiang, Changji Pan, Zhipeng Wang, Yiling Lian, Qingsong Wang, Wenpan Tao, Jingya Sun

**Affiliations:** Laser Micro/Nano Fabrication Laboratory, School of Mechanical Engineering, Beijing Institute of Technology, Beijing 100081, China; College of Control Science and Engineering, China University of Petroleum (East China), Qingdao 266580, China; Yangtze Delta Region Academy of Beijing Institute of Technology, Jiaxing, 314000 Zhejiang, P. R. China; Beijing Institute of Technology Chongqing Innovation Center, Chongqing 401120, China

**Keywords:** double-pulse processing, femtosecond laser, hologram, self-trapped excitons, ultrafast dynamics

## Abstract

Holograms hidden inside transparent materials are important for information encryption storage because of their advantages of secrecy, and could completely avoid information loss caused by surface wear. Inside the transparent material, the modified filaments array was need for hologram fabrication to change the optical phase or amplitude of incident laser, which is sensitive to the change of refractive index. Then the uniformity of modified filaments inside transparent materials is highly required. In this study, by tuning the interval time of the double-pulse processing, holograms with improved imaging quality were fabricated by double-pulse femtosecond laser and the effect and mechanism of self-trapped excitons (STEs) on the ablation have been systematically studied. The imaging quality of the hologram fabricated with double-pulse laser was superior to that of the one fabricated with the single-pulse laser and 350 fs was verified to be the best time interval for double-pulse processing. The evolution of the electrons dynamics was investigated by using the pump-probe technology. With the double-pulse time interval increasing, the residual electrons, excitons, STEs, and defects caused by the first sub-pulse would become dominated sequentially. The results demonstrated the controllability of STEs and quality improvement of final structures by double-pulse femtosecond laser processing.

## 1 Introduction

In recent years, holographic technology has received a considerable interest because of its important application and demand in information storage [1], steganography [2], optical imaging [3], medical imaging [4], and so on. The processing of holograms adopts the method of metasurface fabrication generally via surface plasmon resonance lithography [5], electron/ion beam exposure [6] and femtosecond laser processing [7]. However, lot of holograms fabrications are limited to surface processing, which restrict their wide application. For information storage, especially for information encryption storage, holograms hidden inside materials have great advantages of secrecy, and can completely avoid information loss caused by surface wear. Hologram fabrication usually needs a series of points for the change of the optical phase or amplitude [8–10]. In surface fabrication, array or unit of micro/nano structures such as crater, groove, and hole are required [7, 11]. Inside the transparent material, we need to process the internal structural unit with different refractive index from the raw material, such as modification of filaments, in order to realize the phase delay modulation of the incident laser [10, 12], [13], [14], [15]. Because the phase delay of incident laser is sensitive to the change of refractive index, the uniformity of ablated or modified structures is highly required. Femtosecond laser direct writing and Bessel-shaped processing have been used to process and modify transparent materials for hologram fabrication [12, 13]. However, femtosecond laser direct writing processing needs to move the sample table up and down, which requires high precision of the translation table. And Bessel-shaped processing is easy to damage the surface, which is not conducive to the confidentiality of processing information. In fused silica, the propagation of femtosecond laser is associated with self-focusing and plasma defocusing effect, and the plasma filaments could be formed caused by the balance of the two effects [16, 17]. Then, the modified filaments can be manufactured in fused silica to realize holograms fabrication. This method is convenient and avoids surface damage without moving the sample table up and down. However, it also has disadvantages. As the self-focusing effect is stronger than the plasma defocusing effect at the early stage of the laser propagation, bifurcation is easy to form in the tail of the modified filament, which affects the uniformity of the filament. Thus, it is necessary to find a convenient method to process the modified filaments with great uniformity.

The self-trapped process that electrons, holes or excitons would be trapped in its own lattice deformation when the lattice was not stable on creation of the electron-hole pairs could affect the recombination of the photon generated carriers and the formation of final structure [18–27]. As the product of this process, self-trapped excitons (STEs), were supposed to be the key state during defect formation. STEs could lead directly to the formation of the *E*′ center and defects, which would contribute to the ablation and modification of materials such as void structures formation and changing the refractive index of optical fiber [20, 21, 28], which played an important role in material modification, ablation, and energy relaxation [29–32]. In order to achieve holograms fabrication with great imaging quality, femtosecond laser double-pulse processing was applied to control the STEs and electron ionization, thus improve the uniformity of the modified filaments.

In this study, modified filament arrays were fabricated with single and double-pulse femtosecond laser for holograms application, and 350 fs was verified to be the best time interval for double-pulse processing. The imaging quality of the hologram fabricated with the 350-fs double-pulse laser was superior to that of the one fabricated with the single-pulse laser and the evolution of electron dynamics during the double-pulse femtosecond laser processing of fused silica was analyzed by using pump-probe technology to elucidate the effect of STEs. By adjusting the time interval of the double pulses, the free electron density and exciton state upon the arrival of the second pulse could be controlled; thereby the control of material modification results could be applied. With 350-fs double pulse processing, the laser energy was utilized uniformly, which was attributed to the effects of the STEs and the defect states induced by the first pulse. That is, the presence of the STEs weakened the electron excitation in the early stage of laser propagation and was conducive to the release of pulse energy in the later stage of laser propagation, which indicated the controllability and stability of double-pulse femtosecond laser holograms processing.

## 2 Experimental section

**Figure 1:**
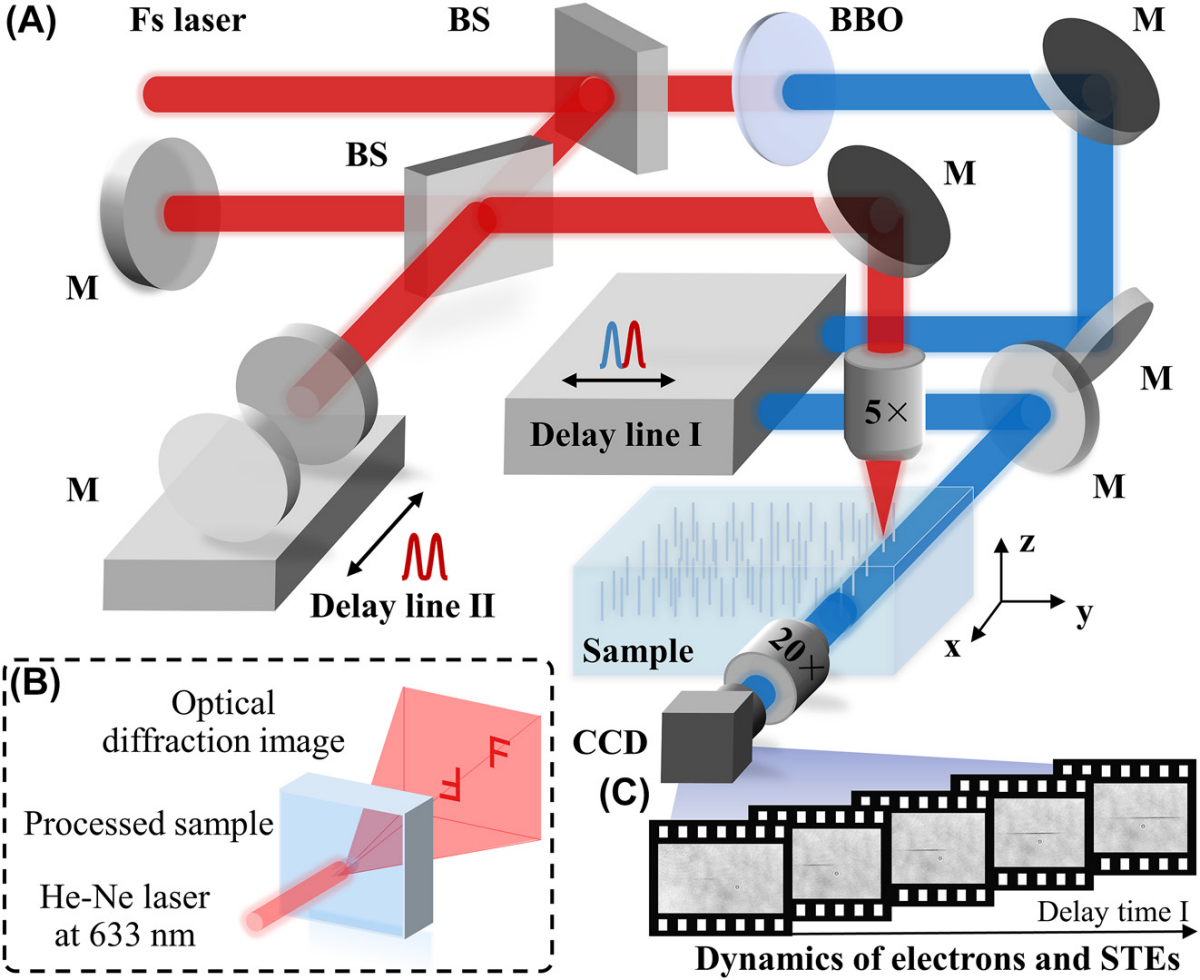
Schematic of experimental setup. (A) The experimental setup schematic of femtosecond laser processing holograms inner fused silica and pump-probe technology. BS: beam splitter coated for 700–1100 nm at 45° incidence, and M: ultrafast mirror. (B) Holograms performance characterization under 633 nm laser illumination. (C) Time-resolved shadowgraphs of the plasma propagation inner fused silica.

The schematics of the experimental setup and results characterization for holograms fabrication and pump-probe technology are shown in Figure 1. Figure 1A depicts the experimental setup for the femtosecond laser holograms processing and pump-probe technology inside fused silica. A femtosecond laser with a pulse duration of 35 fs at 800 nm was divided into two beams that were used as the pump and probe pulses; this was done by using a beam splitter coated for the wavelengths of 700–1100 nm at 45° incidence. The pump beam was divided again by the double-pulse generator (DPG) based on Michelson interferometer to generate double pulses and was finally focused at 300 μm below the sample surface by using a 5 × 0.15 numerical aperture (NA) objective lens (Olympus, Inc.). In order to obtain a spot with high quality, the spot size was limited to 5 mm before focusing. And the spot size after focusing was about 7.6 μm. By controlling the delay line II of the DPG, the time interval of the two pulses was adjusted. On the probe path, the frequency of the probe beam was doubled to 400 nm by using a beta barium borate crystal (BBO), and the beam was placed perpendicular to the pump beam to probe the plasma evolution in the sample. An optical delay line I was used to generate the time delay. Subsequently, the probe beam was passed through the sample, and the time-resolved transmissivity signals were collected using a charge-coupled device (CCD) equipped with a 20 × 0.45 NA objective lens (Olympus, Inc.). The sample was a piece of four-surface polished fused silica measuring 10 × 10 × 1 mm^3^. Figure 1B illustrates the schematic of holograms performance characterization under 633 nm laser illumination. Figure 1C shows the time-resolved shadowgraphs of the plasma propagation and electrons dynamics inner fused silica collected by CCD. To determine the transmissivity change, images were recorded both with and without laser irradiation. In this case, the evolution of the laser-induced plasma and electrons inside the fused silica could be determined in terms of the temporal and spatial resolutions for different double-pulse time intervals.

## 3 Results and discussion

**Figure 2:**
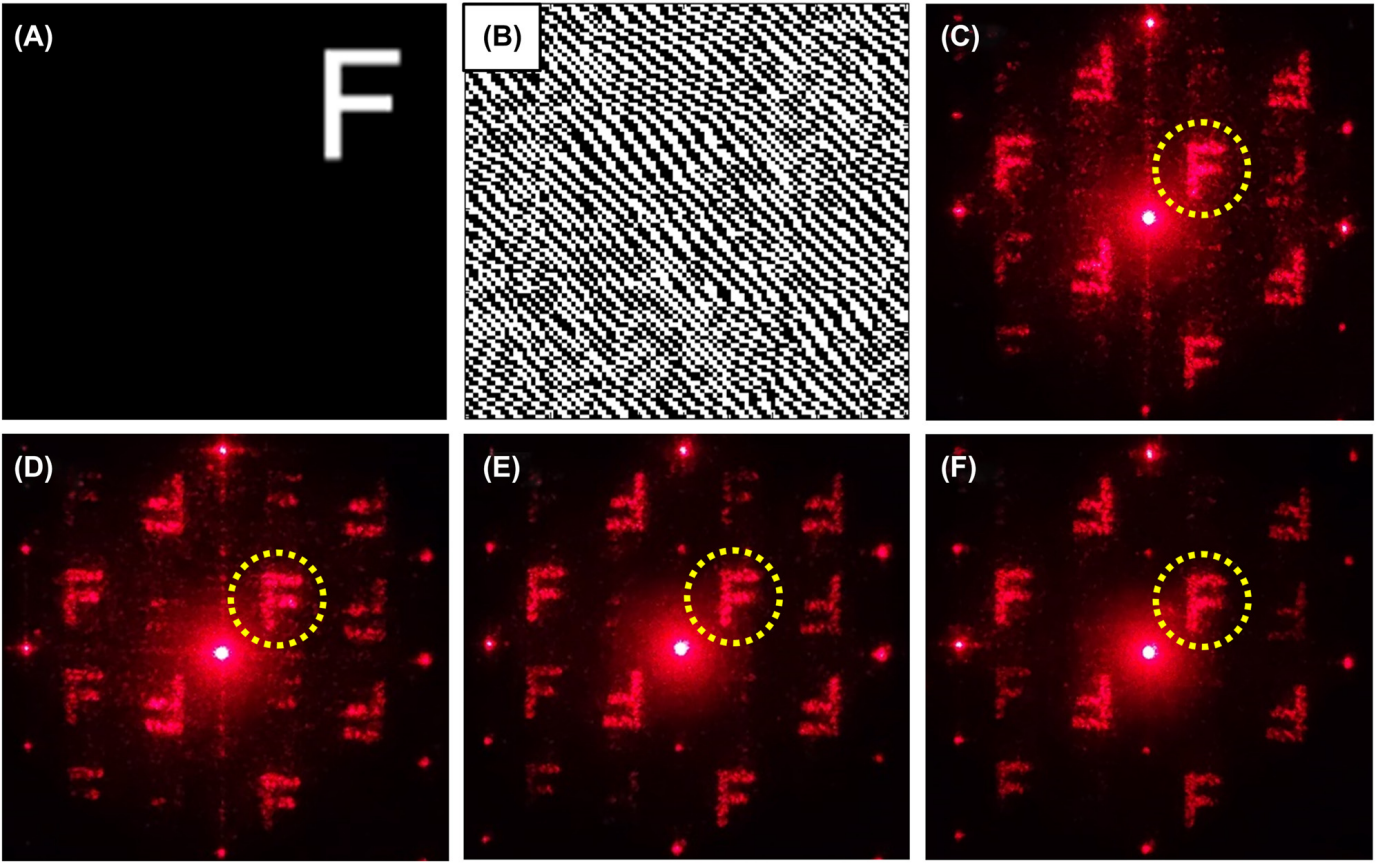
Holograms performance characterization with different double-pulse time intervals. (A) Target reconstruction image, (B) corresponding calculated binary hologram, optical diffraction images reconstructed from the holograms fabricated by single-pulse (C), 150 fs (D), 350 fs (E), and 700 fs (F) double-pulse time intervals, respectively.

Holograms were fabricated by processing the modified filament arrays in fused silica with femtosecond laser single- and double-pulses. Figure 2A and B present the target reconstruction image and the corresponding calculated binary hologram, which is the pattern of the femtosecond laser processing path. The optical diffraction images reconstructed from the holograms fabricated using single- and double-pulse lasers are presented in Figure 2C–F. For single-pulse processing the fluence of pump pulse was 10.6 J/cm^2^, and for double-pulse processing the fluence of each pump pulse was 5.3 J/cm^2^. During femtosecond laser processing, the laser repetition frequency was 1000 Hz, and the irradiation time was 5 s. According to the characteristic time mentioned in previous studies, the electrons-hole recombination time was approximately 150 fs in fused silica, and the sum of the electrons lifetime and free-excitons lifetime was approximately 350 fs [33–37]. Thus, the time interval between the two pulses of the optical diffraction images in Figure 2D–F was selected as 150 fs, 350 fs, and 700 fs, respectively. As shown in the yellow circle of Figure 2C–F, the resolution and signal to noise ratio of ‘*F*’ in Figure 2E were the best, which indicated the imaging quality of the hologram fabricated with the double-pulse laser was obviously superior to that of the hologram fabricated with the single-pulse laser, and the best imaging quality was found in the hologram fabricated with 350 fs time interval.

**Figure 3:**
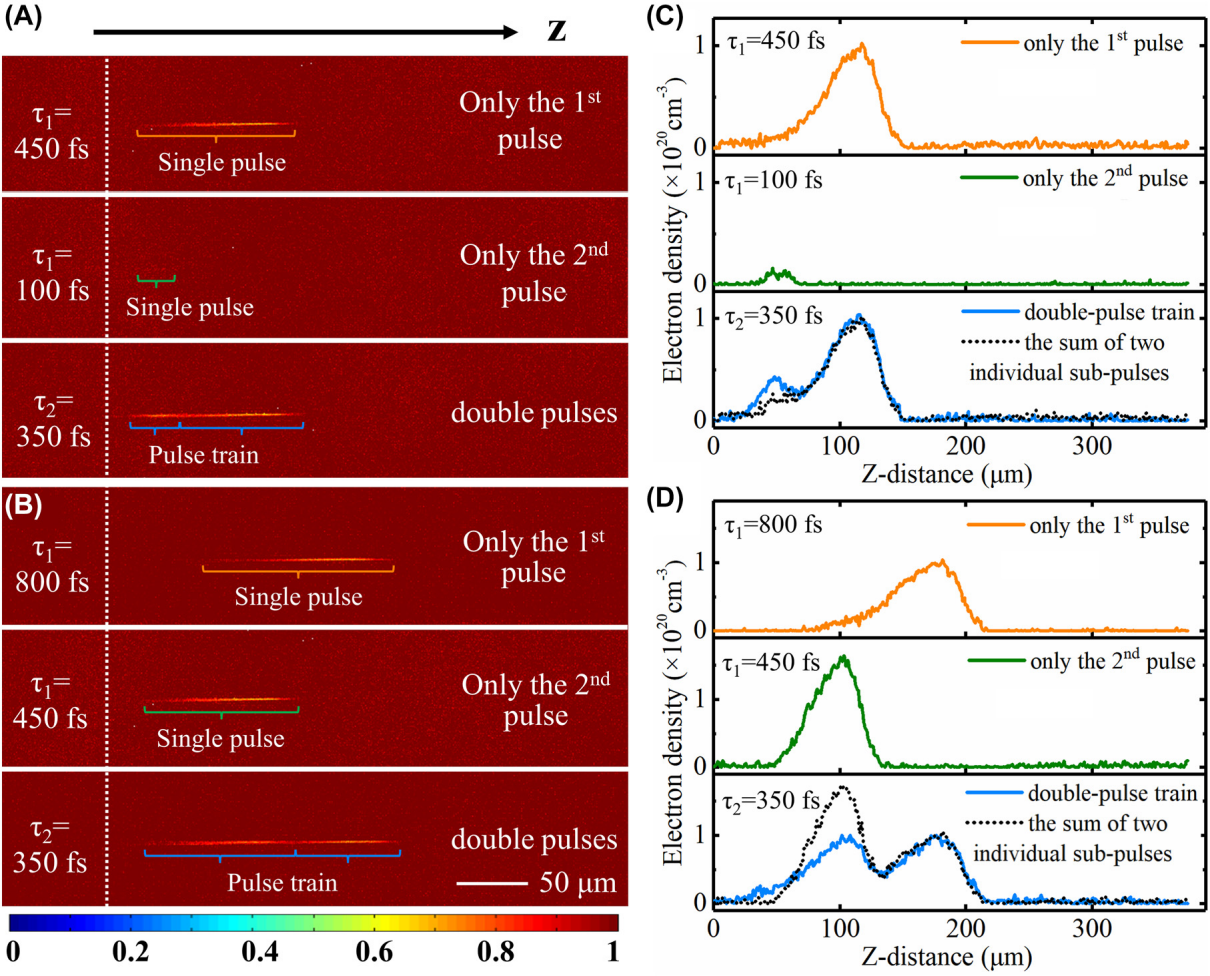
Time-resolved transmissivities of femtosecond laser-induced plasma in fused silica with 350-fs double-pulse time interval (*τ*_2_) and pump-probe delay times (*τ*_1_) of 100 fs (A) and 450 fs (B) relative to the second pulse. The top panel shows the graph with only the first pulse, the middle panel shows the graph with only the second pulse, and the bottom panel shows the graph with both pulses. (C, D) Evolution of electron density corresponding to (A, B). The fluence of each pump pulse was 5.3 J/cm^2^.

To explain the holograms imaging improvement with double-pulse femtosecond laser processing, the temporal and spatial evolution of electron density was recorded at the double-pulse time intervals of 150, 200, 250, 300, 350, 400, 500, 600, and 700 fs. 150, 350, and 700 fs were selected as the characteristic double-pulse time intervals in the main text to investigate the temporal and spatial evolution of electron density. The experiments data of the other cases were showed in Figures S1–S4 in Supporting Information. As a typical condition, the time-resolved transmissivities of femtosecond-laser-induced plasma with the double-pulse time intervals of 350 fs are shown in Figure 3. Figure 3A and B show the experimental results with the probe delay times of 100 fs and 450 fs, respectively. To make a clear distinction about the time intervals mentioned above, *τ*_1_ and *τ*_2_ were used to present the pump-probe delay time relative to the second pump pulse and the double-pulse time interval respectively in Figure 3. Figure 3C and D present plots of the spatial distribution of electron density obtained along the center axis of the laser-induced plasma filament. In order to compare the situation of two sub-pulses irradiated simultaneously and separately in a double pulse, the top and middle rows of each figure represent cases of irradiation with only the first and only the second pump pulses, respectively. The bottom row represents the case of irradiation with both pulses, and the black dotted line represents the superimposition of the electron density data in the top and the middle rows. The delay time relative to the second and first sub-pulse was 100 fs, 450 fs, and 450 fs, 800 fs because of the 350-fs interval of double pulses in Figure 3A and C and Figure 3B and D. The method used to extract the electron density *n*_*e*_ was that used in previous studies [17, 38], [39], [40]. Briefly, based on Drude model, the dielectric function *ε* could be described as *ε* = *ε*_0_ − *n*_*e*_ *e* ^2^/[*m*_*e*_ *ε*_0_(*ω* ^2^ + i*ω*/*τ*_*e*_)], where *ε*_*0*_ *and ω* are the permittivity of the vacuum and laser frequency; *m*_*e*_ and *e* are the mass of the electron and electric quantity; and *τ*_*e*_ is electron scattering time which is chosen as 0.2 fs [40]. Meanwhile, *ε* was related to the absorption coefficient *α* with *α* = 4*π*Im(*ε* ^1/2^)/*λ*, and transmissivity *T* can be described as *T* = *I*_*t*_/*I*_0_ = *e* ^−*αz*^ *.* Therefore, *n*_*e*_ was associated with the optical parameter *T* [40, 41]. It can be seen from Figure 3C and D that the second sub-pulse of the double pulses played a significant role on the spatial distribution of free electron density. In the early stage of femtosecond laser propagation, such as the 100 fs probe time delay, the second sub-pulse showed a positive effect on electron ionization. However, with the delay time increasing, such as the 450 fs probe time delay, the electron density for the double-pulse irradiation case was lower than that of the data superposition with single pulse irradiation.

**Figure 4:**
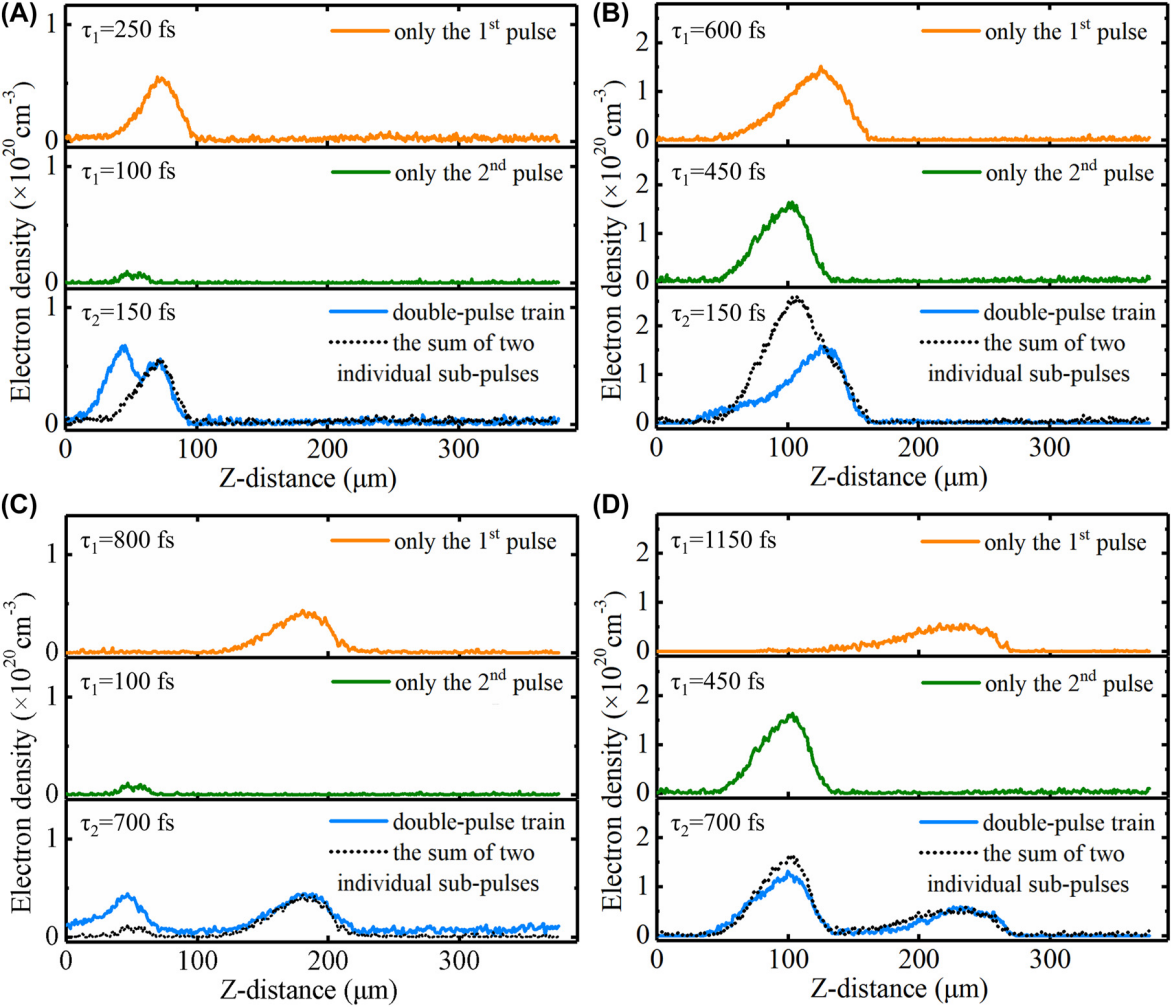
Electron density evolution with pump-probe delay times (*τ*_1_) of 100 (A, C) and 450 (B, D) fs relative to second pulse. The top panel depicts the graph obtained for irradiation with only the first pulse, the middle panel depicts the graph obtained for irradiation with only the second pulse, and the bottom panel depicts the graph obtained for irradiation with both pulses. (A, B) and (C, D) depict the cases with the double-pulse time intervals (*τ*_2_) of 150 and 700 fs, respectively.

In the cases of the other two double-pulse intervals, the spatial distributions of electron density for different probe delay times of 100 and 450 fs were shown in Figure 4. Specifically, Figure 4A and B show the cases with the 150-fs double-pulse time interval, and Figure 4C and D show the cases with the 700-fs double-pulse time interval. Similar to the phenomenon depicted in Figure 3, the electron density after excitation with the double pulses was higher than that after excitation with only a single pulse in the early stage of laser propagation (100 fs probe time delay), and then decreased as the delay time increased (450 fs probe time delay). Generally, the first sub-pulse would excite the electrons from valence band to conduction band and then electron recombination occurred and excitons and STEs began to form. For the double-pulse excitation in the early stage (100 fs probe delay time), the second sub-pulse propagated in the sample and the electron excitation would occur more easily because of the presence of the residual electrons excited by the first sub-pulse, excitons and STEs [42]. Thus, the electron density was higher than that excited without the first pulse irradiation. However, with the longer propagation time (450 fs probe delay time), the generation of more residual electrons by the first sub-pulse could cause the electron screening, so that the energy absorption of the second sub-pulse would be blocked [43, 44]. Thus, it was harder for the pulse to propagated inward, which showed that the electron density excited by the second pulse decreased compared with that after excitation by only a single pulse.

**Figure 5:**
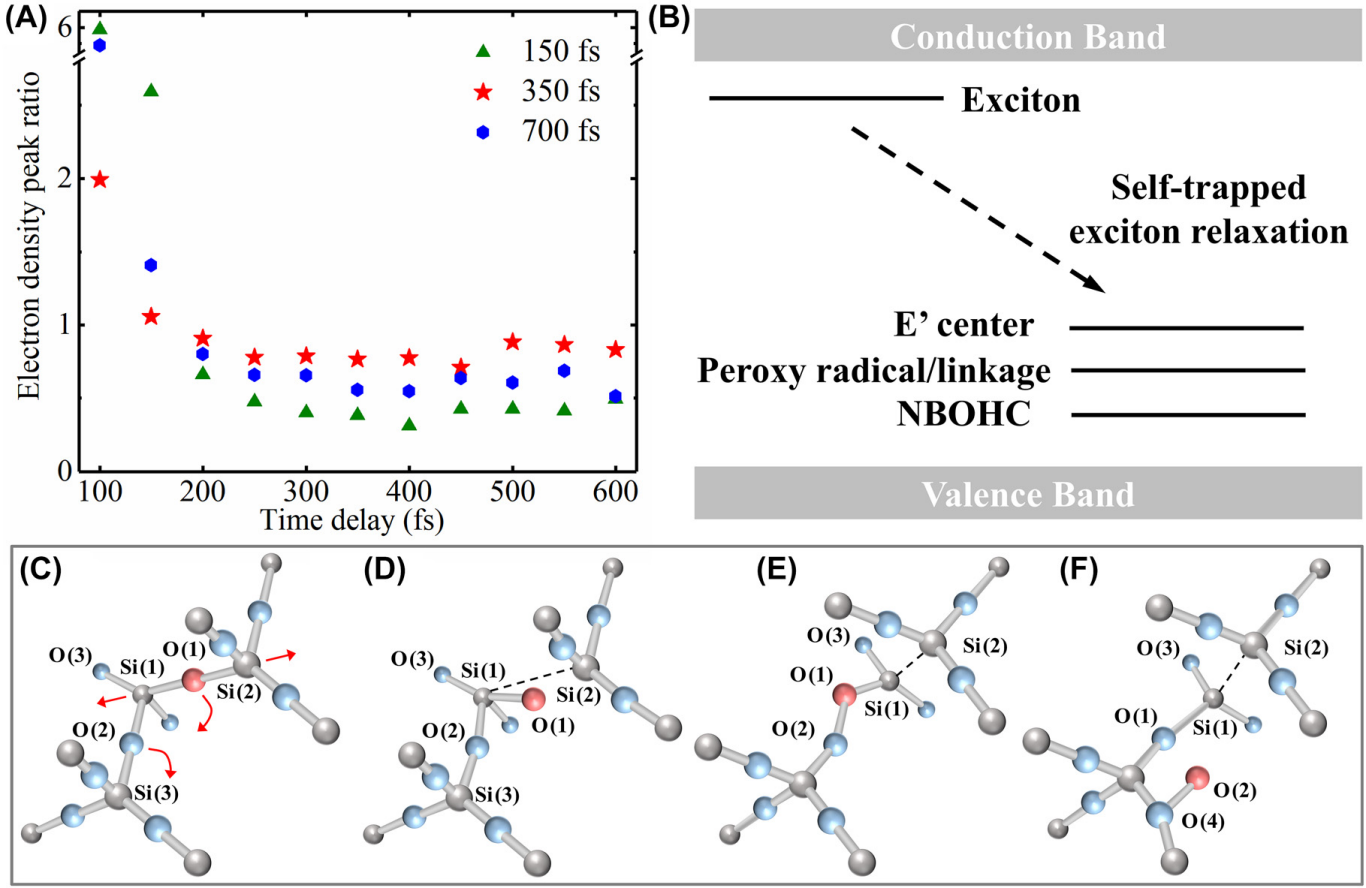
The effect of self-trapped exciton on free electron density. (A) Evolution of electron density peak ratio (*n*_e-max_ with double-pulse irradiation and *n*_e-max_ superimposed with single-pulse irradiation data) with pump-probe delay time for double-pulse irradiation with time intervals of 150, 350, and 700 fs. (B) Schematic of exciton and defect energy levels in fused silica. (C) Fragment of the lattice structure before displacement, with the arrows showing the directions of the atomic relaxations. (D) Equilibrium configuration of STEs. (E) Nearest-neighbor defect pair (peroxy linkage [Si–O–O–Si]). (F) Separated defect (peroxy radical [Si–O–O•]).

As shown in Figures 3 and 4, the electrons excited by the above double pulses with three different time intervals exhibited similar evolutionary trends at 100 fs and 450 fs probe time. In order to investigate the details of the electron evolution, the electron density peak ratio with the probe delay time from 100 fs to 600 fs were extracted and shown in Figure 5A. The electron density peak ratio was the ratio of *n*_e-max_ of data with double-pulse irradiation and *n*_e-max_ of superimposed data with single-pulse irradiation. For clarity, Figure 5 showed the cases with the double pulse time intervals of 150, 350, and 700 fs, and the other cases were shown in Figure S5. With all time intervals double-pulse irradiation, the electron density peak ratio was greater than 1 at the beginning and then turned to less than 1. In the case of double-pulse irradiation with the time interval of 350 fs, the electron density ratio was relatively lower in the early stage of propagation of the second pump pulse and then increased to a higher value than those in the other cases with laser propagating, which meant that the trend of the electron density peak ratio was relatively more stable with 350-fs double-pulse irradiation. To explain the detailed mechanisms during the electrons dynamics evolution, Figure 5B schematically illustrates the energy levels of exciton, STEs and defects in fused silica. After irradiation with the first sub-pulse, the electrons were excited from the valence band to the conduction band, and they started to recombine and diffuse [45–48]. The lifetime of these free electrons was approximately 150 fs. However, not all of the free electrons recombined with the holes in the valence band of fused silica. An electron and a hole can bind together to form an electrically neutral exciton through Coulomb attraction [49]. These excitons are unstable and can relax through localized channels in wide bandgap dielectrics, as indicated by the dotted arrow in Figure 5B. Then self-trapping can occur because of the small atomic displacement, and free excitons can be trapped to form STEs through their interactions with lattice distortions [36]. Corresponding to energy levels of different states in Figure 5B, Figure 5C–F show the lattice structures in different stages after femtosecond laser irradiation. The lattice structure before displacement is shown in Figure 5C, where the arrows indicate the directions of the atomic relaxations. The free-excitons relaxation process lasted approximately 250 fs, as measured in previous studies [34, 50]. Subsequently, the oxygen vacancy trapped a hole and left an unpaired electron in the adjacent silicon atom to form the *E*′ center (Si•) and the nonbridging oxygen hole centers (NBOHC, Si–O•), as illustrated in Figure 5D. The displaced oxygen atom occupied an interstitial site and could possibly end up in a peroxy linkage [Si–O–O–Si] or radical [Si–O–O•], as depicted in Figure 5E and F [36, 51].

In our experiment, when the double-pulse time interval was 150 fs, the electrons excited by the first pump pulse were mostly transformed into excitons and recombined with the holes in the valence band in small amount. The energy level of the excitons was close to the conduction band, meaning that the excitons were easily excited by the second pulse to form a large number of free electrons. When the double-pulse time interval increased to 350 fs, the excitons induced by the first pulse were self-trapped by the deformed lattice. That is, the free-excitons were relaxed to form the *E*′ center and the NBOHC, and their energy levels were lower than that of the excitons. Thus, it was more difficult to excite the free electrons to the conduction band than it was in the case with the double-pulse time interval of 150 fs. The electron density peak ratio was lower in this case. When the double-pulse time interval was 700 fs, the peroxy linkage and radicals were formed in large numbers. In this case, the number of unpaired electrons on the dangling bond of Si was higher than that of STEs, as illustrated in Figure 5D–F. It was easier for the second pulse to excite the electrons in the peroxy linkage and radicals. As the probe delay time increased, the electron density excited in the cases of 150 and 700 fs was lower than that in the case of 350 fs, which was ascribed to obstruction of energy absorption caused by the electron screening in the early stage. Thus, when the double-pulse time interval was 350 fs, the energy of the second pulse was used more evenly in the process of laser propagation.

**Figure 6:**
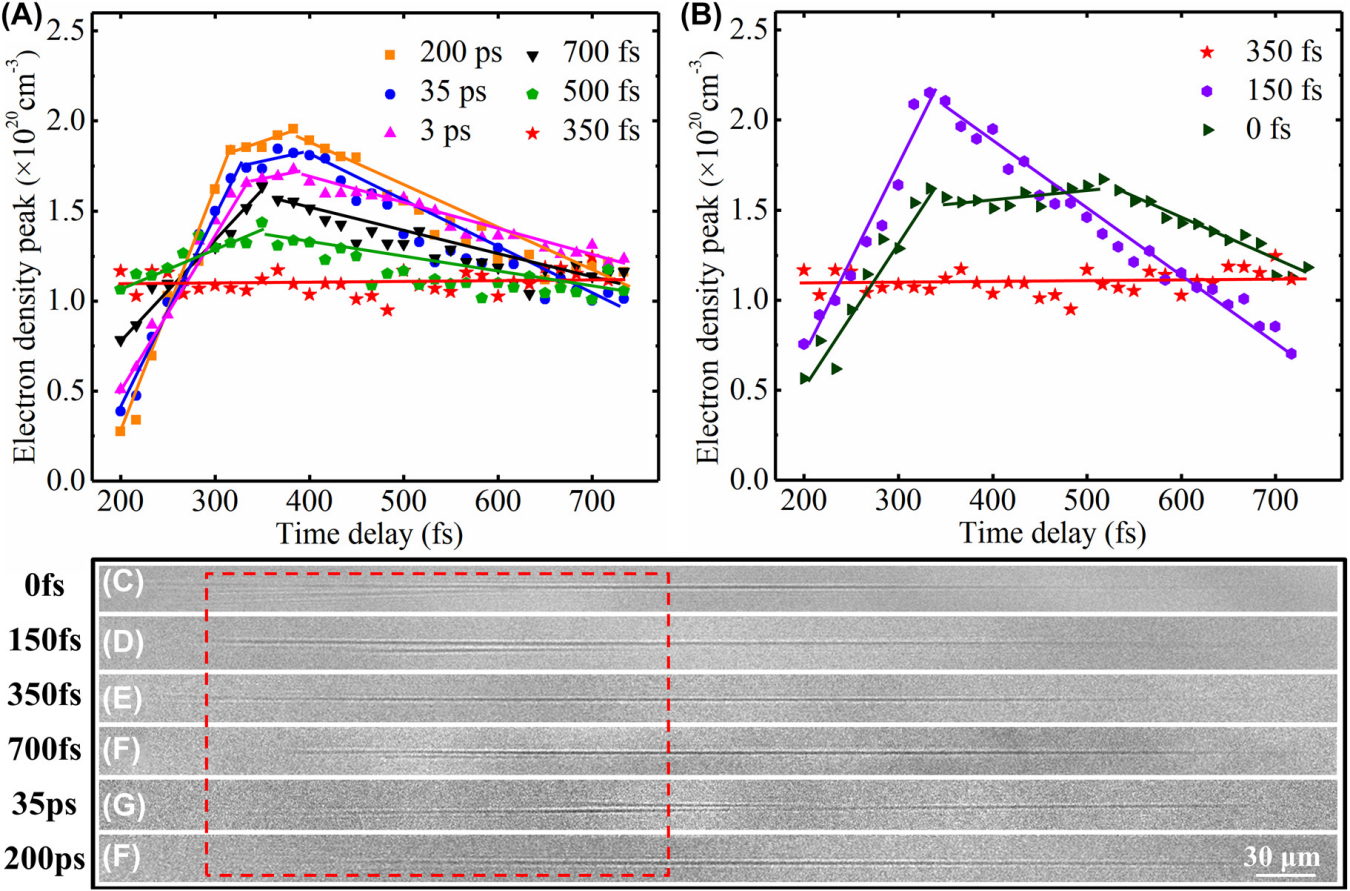
Electron density peak and modified filaments inner fused silica with different double-pulse time intervals. (A, B) Electron density peak for pump-probe delay times of 200–750 fs with double-pulse time intervals ranging from 0 to 200 ps. (C–H) The modified filaments inner fused silica induced by the single-pulse and double-pulse femtosecond laser with time intervals from 0 fs to 200 ps. The laser repetition frequency is 1000 Hz, and the pulse duration is 5 s.

The peak electron density was indicated to be equally important because it was associated with the ablation state. It is widely acknowledged that when the free electron density reaches the critical electron density, ablation commences [52–55]. Because of the relevance between the electron density peak and the ablation state, the evolution of the electron density peak with time delay is shown in Figure 6. Figure 6A and B depict the electron density peaks picked at pump-probe delay times of 200–750 fs with different double-pulse time intervals. Figure 6A shows the cases with 350 fs, 500 fs, 700 fs, 3 ps, 35 ps, and 200 ps double-pulse time intervals and Figure 6B shows the cases with 0 fs, 150 fs, and 350 fs double-pulse time intervals, where the points denote the experimental data and the lines are marked for clarity. Throughout the laser propagation process, the electron density peak corresponding to the double-pulse time interval of 350 fs remained stable at approximately 1.1 × 10^20^ cm^−3^. Although the electron density peaks corresponding to the other time intervals were higher than that corresponding to 350 fs, their fluctuations were higher, which was not conducive to achieving the uniformity of processing. In order to prove the conjecture about the relationship between electrons density evolution and processing uniformity, Figure 6C–H illustrate the modified filaments inside the fused silica induced by the single- and double-pulse femtosecond lasers. The best processing quality was achieved with 350-fs double-pulse irradiation, as indicated in Figure 6E. Especially as shown from the tail of the filaments (the red square in Figure 6C–H), bifurcation could be obviously observed from the filaments fabricated with 0, 150, and 700 fs, and 35, 200 ps. For 350-fs double-pulse irradiation, the refractive index of this filament was relatively uniform, and bifurcation was absent at the tail, which was attributed to the stable evolution of the electron density peak. In this case, STEs decelerated the excitation of electrons in the initial stage of laser propagation and aided the release of laser energy during the later stage of laser propagation. This effect improved the uniformity of laser energy utilization during pulse propagation and thus improved the processing quality of the modified filament. Thus, the main reason for higher imaging quality of holograms in Figure 2 was attributed to the uniform refractive index of filaments induced by ionization controlling based on the self-trapped excitons with double-pulse femtosecond-laser. This result highlights the electrons dynamics control of in double-pulse hologram processing and thus confirms the effects of STEs on material modification and ablation.

In conclusion, holograms with improved imaging quality were fabricated by processing modified filament arrays in fused silica with femtosecond laser double-pulse fabrication, and the electron dynamics evolution and the effect of STEs on the femtosecond laser double-pulse processing of fused silica were investigated in a pump-probe experiment. The time interval of the double pulses was adjusted to control the free electron density and exciton state upon the arrival of the second pulse, which aided the control of the electrons dynamics and material modification results following processing with the second pulse. The time interval of 350 fs between the two pulses, which corresponded to the total time of free electron decay and exciton trapping, was verified to be the best time interval for double-pulse processing and holograms imaging quality. These results indicated the key effects of the STEs on material modification and ablation, and demonstrated the controllability and superiority of double-pulse femtosecond laser processing based on electrons dynamics control.

## Supplementary Material

Supplementary Material Details
