# Supplementary material for: Hologram imaging quality improvement by ionization controlling based on the self-trapped excitons with double-pulse femtosecond laser
Source: Nanophotonics. 2022 Oct 24;11(21):4727–36. doi: 10.1515/nanoph-2022-0379 (PMC11501880; doi:10.1515/nanoph-2022-0379)
Supplement: Supplementary file 1 — Supplementary Material Details [file j_nanoph-2022-0379_suppl.docx]

**Supplementary Material**

**Hologram imaging quality improvement by ionization controlling based on the self-trapped excitons with double-pulse femtosecond laser**

Feifei Wang^1^, Lan Jiang^1,2,3^, Changji Pan^1^, Zhipeng Wang^1^, Yiling Lian^1^, Qingsong Wang^1^, Wenpan Tao ^1^ and Jingya Sun*^1,2^

*^1^ Laser Micro/Nano Fabrication Laboratory,* *School of Mechanical Engineering, Beijing Institute of Technology, Beijing 100081, China*

*^2^ Yangtze Delta Region Academy of Beijing Institute of Technology, Jiaxing, 314000 Zhejiang, P.R. China*

*^3^ Beijing Institute of Technology Chongqing Innovation Center, Chongqing 401120, China*

*E-mail: sjy@bit.edu.cn

1. **The temporal and spatial evolution of electron density in fused silica**

The temporal and spatial evolution of electron density in fused silica was recorded at the double-pulse time intervals of 150, 200, 250, 300, 350, 400, 500, 600 and 700 fs. The experiments data of the all cases were showed in Figure S1–S4.


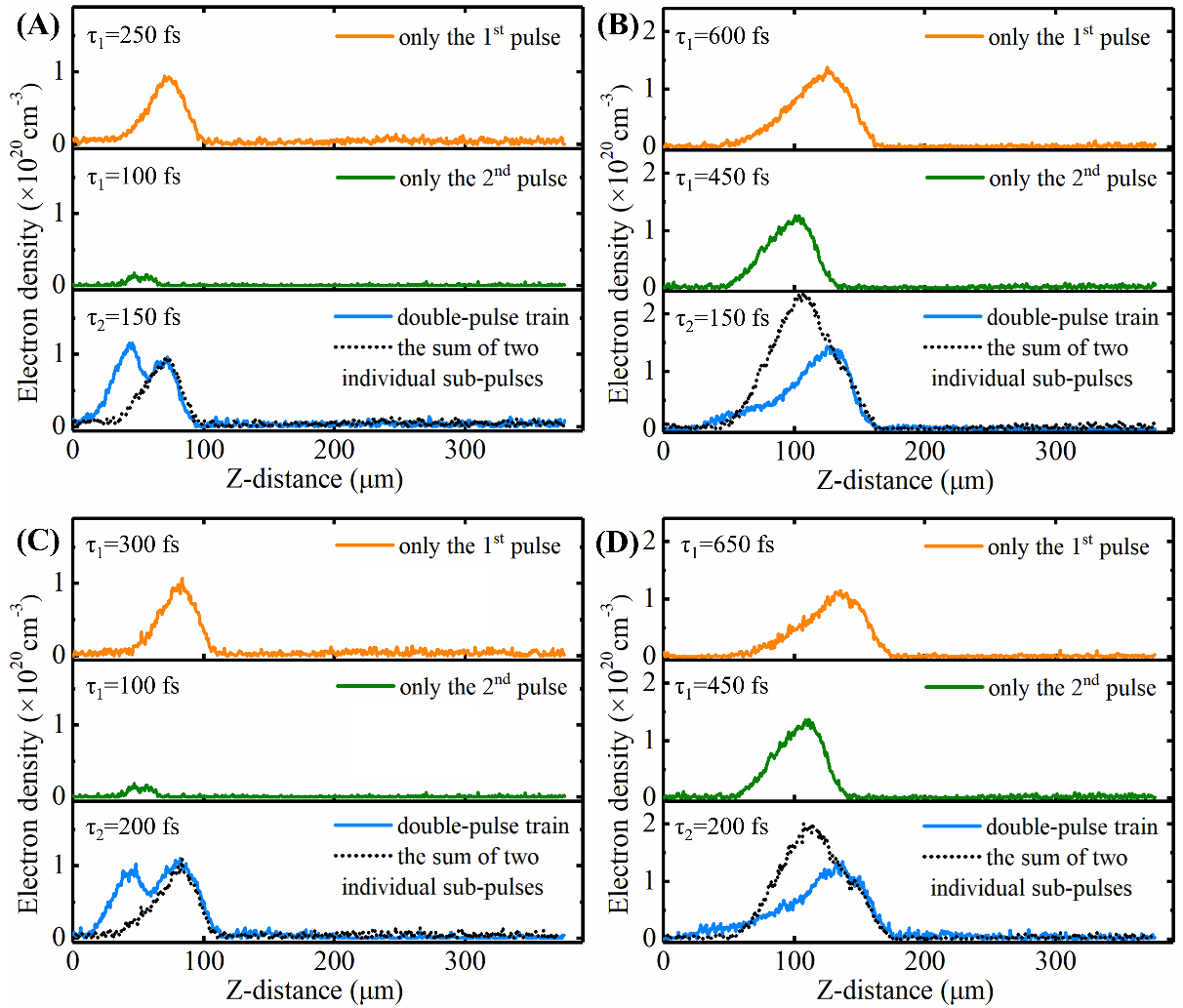


**Figure S1.** Electron density evolution with pump-probe delay times (τ_1_) of 100 (A, C) and 450 (B, D) fs relative to second pulse. The top panel depicts the graph obtained for irradiation with only the first pulse, the middle panel depicts the graph obtained for irradiation with only the second pulse, and the bottom panel depicts the graph obtained for irradiation with both pulses. (A, B) and (C, D) depict the cases with the double-pulse time intervals (τ_2_) of 150 and 200 fs, respectively.


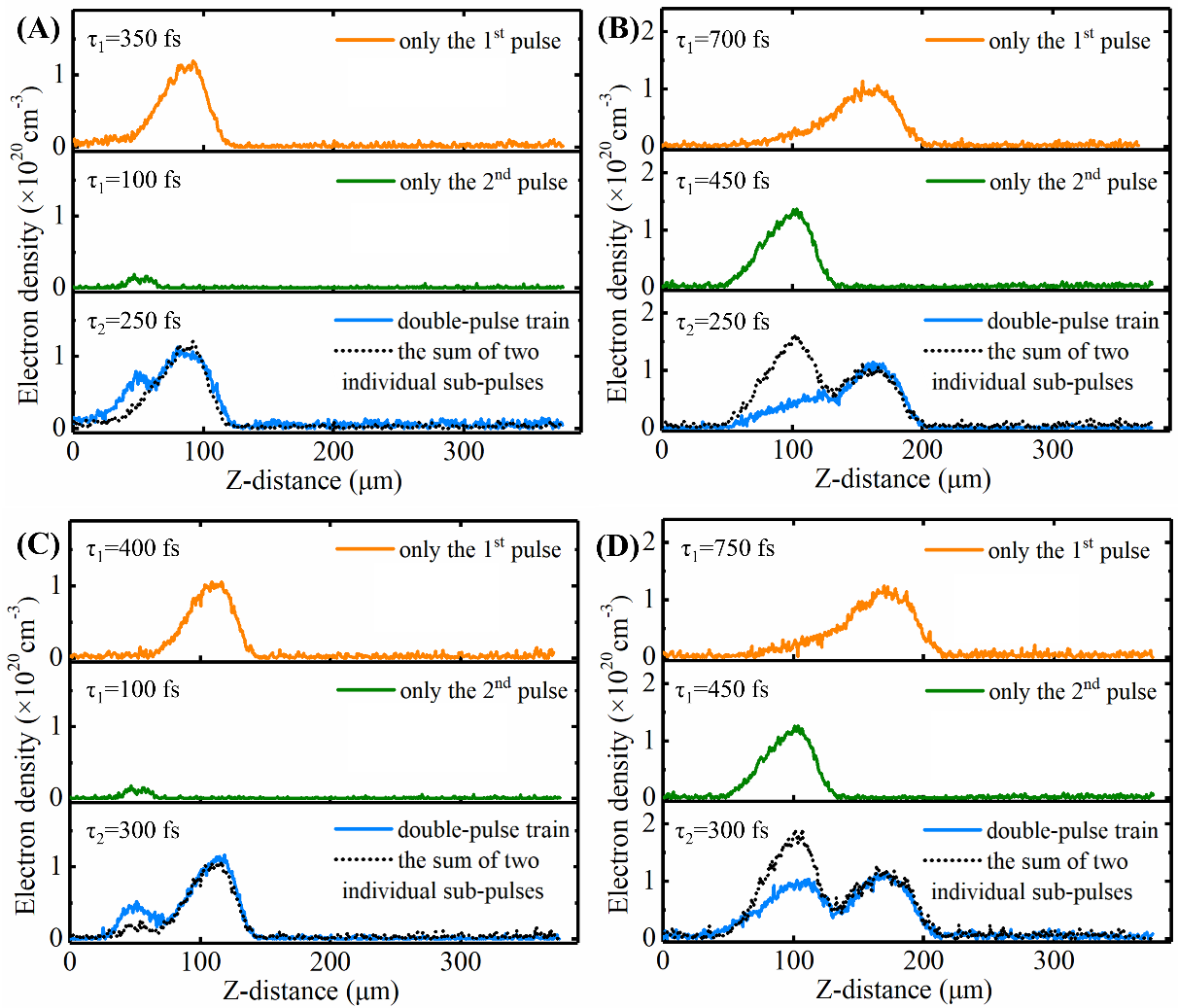


**Figure S2.** Electron density evolution with pump-probe delay times (τ_1_) of 100 (A, C) and 450 (B, D) fs relative to second pulse. (A, B) and (C, D) depict the cases with the double-pulse time intervals (τ_2_) of 250 and 300 fs, respectively.


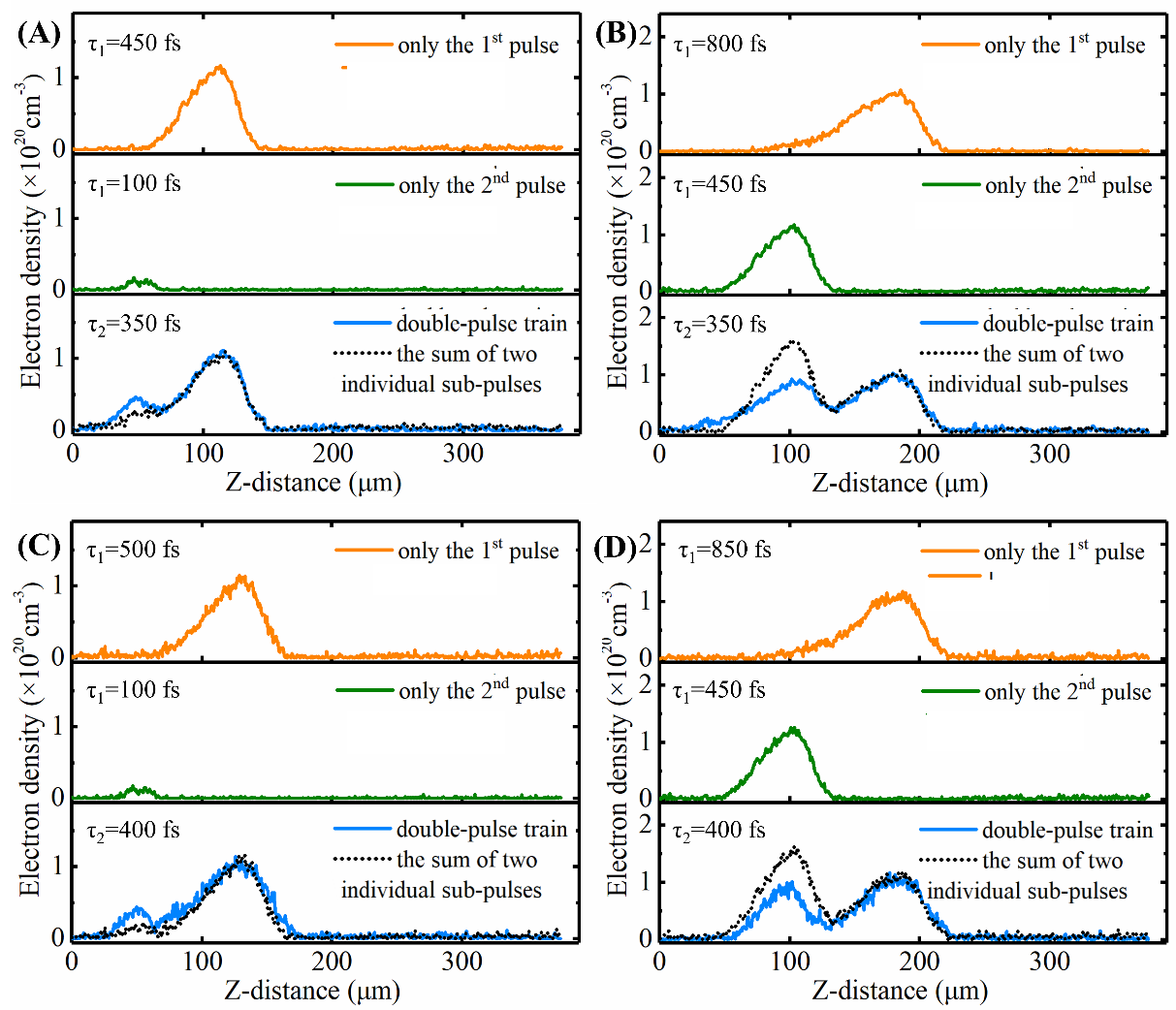


**Figure S3.** Electron density evolution with pump-probe delay times (τ_1_) of 100 (A, C) and 450 (B, D) fs relative to second pulse. (A, B) and (C, D) depict the cases with the double-pulse time intervals (τ_2_) of 350 and 400 fs, respectively.


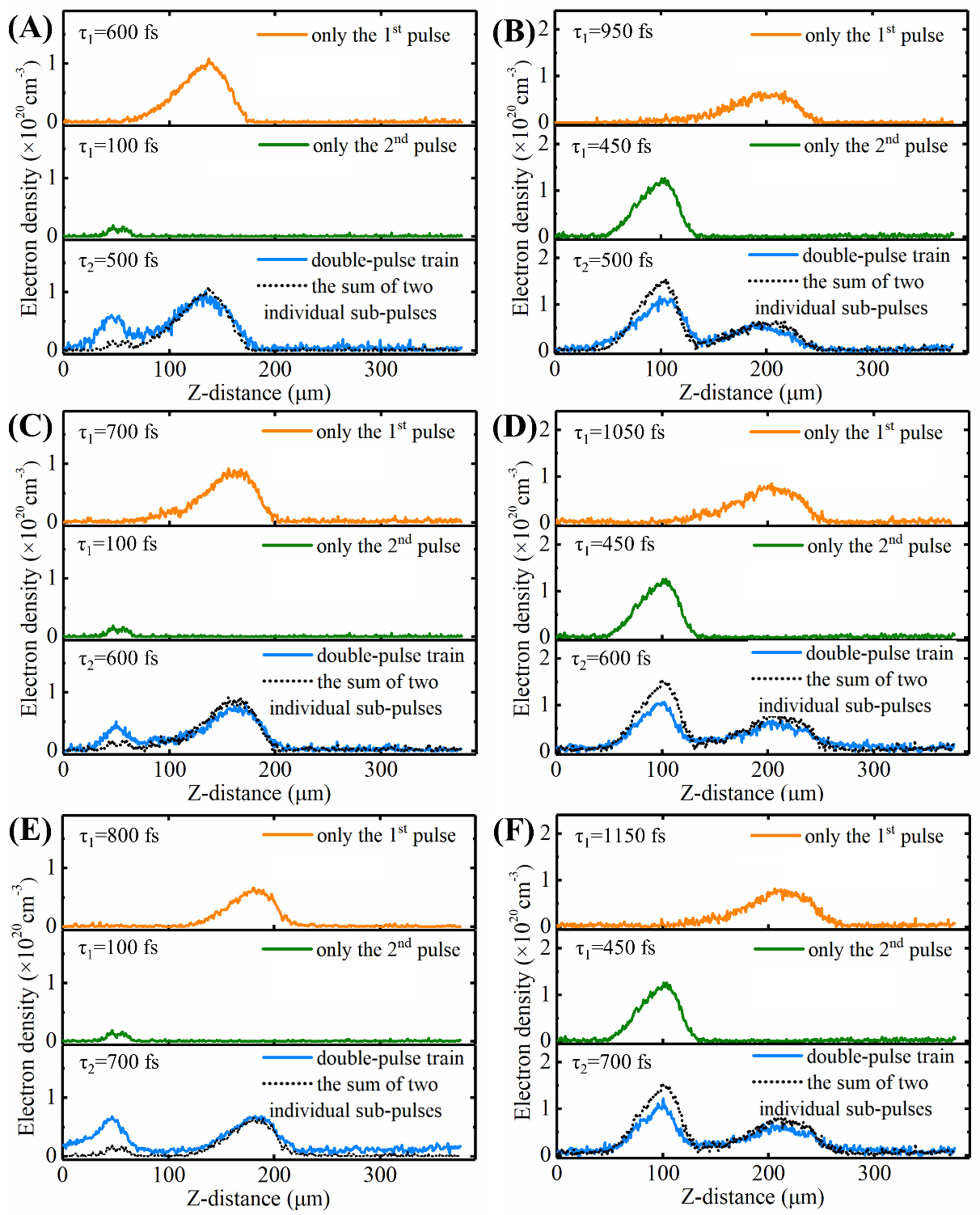


**Figure S4.** Electron density evolution with pump-probe delay times (τ_1_) of 100 (A, C, E) and 450 (B, D, F) fs relative to second pulse. (A, B), (C, D) and (E, F) depict the cases with the double-pulse time intervals (τ_2_) of 500, 600 and 700 fs, respectively.

1. **Evolution of electron density peak ratio**

In order to investigate the details of the electron evolution, the electron density peak ratio with the probe delay time from 100 fs to 600 fs were extracted and shown in Figure S5. The electron density peak ratio was the ratio of n_e-max_ of data with double-pulse irradiation and n_e-max_ of superimposed data with single-pulse irradiation. Figure S5 showed the cases with the double pulse time intervals of 150, 200, 250, 300, 350, 400, 500, 600 and 700 fs.





**Figure S5.** Evolution of electron density peak ratio (n_e-max_ with double-pulse irradiation and n_e-max_ superimposed with single-pulse irradiation data) with pump-probe delay time for double-pulse irradiation with time intervals of 150, 200, 250, 300, 350, 400, 500, 600 and 700 fs.
